# Efficient Self-Assembly of mPEG End-Capped Porous Silica as a Redox-Sensitive Nanocarrier for Controlled Doxorubicin Delivery

**DOI:** 10.1155/2018/1575438

**Published:** 2018-03-01

**Authors:** Anh Khoa Nguyen, Thi Hiep Nguyen, Bui Quoc Bao, Long Giang Bach, Dai Hai Nguyen

**Affiliations:** ^1^Graduate University of Science and Technology, Vietnam Academy of Science and Technology, Hanoi 100000, Vietnam; ^2^Institute of Applied Materials Science, Vietnam Academy of Science and Technology, 01 TL29, District 12, Ho Chi Minh City 700000, Vietnam; ^3^Tra Vinh University, No. 126, Nguyen Thien Thanh, Ward 5, Tra Vinh City, Tra Vinh Province 940000, Vietnam; ^4^Tissue Engineering and Regenerative Medicine Group, Department of Biomedical Engineering, International University, Vietnam National University-HCMC (VNU-HCMC), Ho Chi Minh City 70000, Vietnam; ^5^Nguyen Tat Thanh University, 300A Nguyen Tat Thanh, District 4, Ho Chi Minh City 700000, Vietnam

## Abstract

Porous nanosilica (PNS) has been regarded as a promising candidate for controlled delivery of anticancer drugs. Unmodified PNS-based nanocarriers, however, showed a burst release of encapsulated drugs, which may limit their clinical uses. In this report, PNS was surface conjugated with adamantylamine (ADA) via disulfide bridges (-SS-), PNS-SS-ADA, which was further modified with cyclodextrin-poly(ethylene glycol) methyl ether conjugate (CD-mPEG) to form a core@shell structure PNS-SS-ADA@CD-mPEG for redox triggered delivery of doxorubicin (DOX), DOX/PNS-SS-ADA@CD-mPEG. The prepared PNS-SS-ADA@CD-mPEG nanoparticles were spherical in shape with an average diameter of 55.5 ± 3.05 nm, a little larger than their parentally PNS nanocarriers, at 49.6 ± 2.56 nm. In addition, these nanoparticles possessed high drug loading capacity, at 79.2 ± 3.2%, for controlled release. The release of DOX from DOX/PNS-SS-ADA@CD-mPEG nanoparticles was controlled and prolonged up to 120 h in PBS medium (pH 7.4), compared to less than 40 h under reducing condition of 5 mM DTT. Notably, the PNS-SS-ADA@CD-mPEG was a biocompatible nanocarrier, and the toxicity of DOX was dramatically reduced after loading drugs into the porous core. This redox-sensitive PNS-SS-ADA@CD-mPEG nanoparticle could be considered a potential candidate with high drug loading capacity and a lower risk of systemic toxicity.

## 1. Introduction

Over the last several years, an increased amount of porous nanosilica (PNS) has been designed and developed owing to its high biocompatibility and biodegradability, tunable pore size, controllable morphology and easily modifiable surfaces, high chemical and thermal stabilities, straightforward preparation, and ability to carry various guest molecules with the porous pores [1–4]. The unique structure of PNS allows it to effectively encapsulate different guest molecules within the pores and protect them from enzymatic degradation [5–7]. However, encapsulated drugs would burst release from the unmodified PNS nanoparticles, leading to the loss of drugs that reach cancerous cells. In order to overcome this limitation, multifunctional polymer-modified PNS is one of the most critical insights; both interfacial properties of the modified nanocarriers can be engineered and thermal and mechanical features of polymers can be improved at the same time. Nevertheless, this preparation can control and delay the rate of drug release instead of deciding when or where to deliver drugs [7, 8].

Recently, stimuli-sensitive PNS has attracted enormous interests due to its ability to respond to both internal stimuli, such as pH, redox potential, and temperature, and external stimuli, including light, magnetic fields, and ultrasound. Therefore, encapsulated drugs can be triggered by using appropriate stimuli [9–13]. The redox stimulus among those stimuli is one of the most effective strategies because the concentration of glutathione (GSH) in cancer cells (2–10 mM) is 100 to 1000-fold higher than that in normal healthy cells (1-2 *μ*M). This phenomenon could offer an opportunity for redox-responsive system to release anticancer drugs at the targeted tumor sites, in order words [14–16]. For instance, Wang et al. modified PNS with polyethylene glycol (PEG) through disulfide bonds to achieve on-demand controlled release of a model drug rhodamine B (RhB). The results of* in vitro* assay showed that RhB was released dramatically under 10 mM GSH condition. Plus, the biocompatibility of the modified PNS nanoparticles increased with increasing surface PEG density [17, 18]. Wang and coworkers also developed a redox and pH dual-responsive nanocarrier for site-specific drug delivery, in which poly(acrylic acid) (PAA) was grafted in the outlets of PNS through cleavable disulfide bonds. RhB was also significantly released in GSH condition or pH 5.0 PBS. This PNS-SS-PAA nanoparticle exhibited dual-responsive drug release property and can be further used as a promising candidate for the treatment of cancer [19]. The complex structure of modified PNS delivery systems, in particular, was capable of loading high levels of drugs. Notably, if the pore entrances of PNS nanosystems have firstly been blocked by caps before drug encapsulation, an amount of nonencapsulated drug would increase [7].

**Figure 1:**
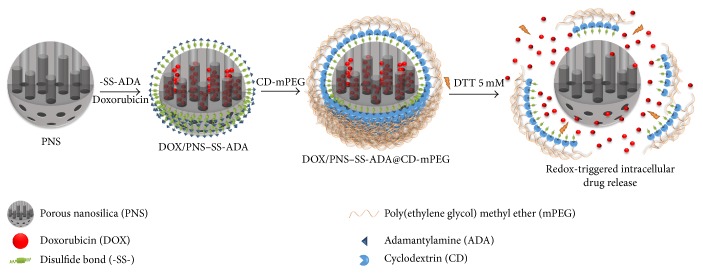
Schematic showing the formation of redox-responsive DOX/PNS-SS-ADA@CD-mPEG nanocarriers.

In this study, we prepared potential PNS-based redox-responsive nanocarriers for controlled drug release. The surface of PNS was first modified with adamantylamine (ADA) via disulfide linkages (-SS-) and further functionalized with cyclodextrin-poly(ethylene glycol) methyl ether conjugate (CD-mPEG) via a strong complexation of ADA and CD, PNS-SS-ADA@CD-mPEG. Furthermore, DOX was used to evaluate drug loading into the PNS-SS-ADA@CD-mPEG nanoparticles (Figure 1). The obtained samples were characterized by proton nuclear magnetic resonance (^1^H NMR), Fourier transform infrared (FTIR), thermogravimetric analysis (TGA), N_2_ adsorption-desorption (BET), and transmission electron microscope (TEM). Especially, dithiothretol (DTT) was used to reduce –SS- in PNS-SS-ADA@CD-mPEG. Additionally, MTT assay was performed to determine whether the PNS-SS-ADA@CD-mPEG nanoparticle may reduce the toxicity to HeLa cells of DOX. This study is expected to create a redox-sensitive nanocarrier with high drug loading capacity and low systemic toxicity for controlled drug delivery.

## 2. Materials and Methods

### 2.1. Materials

Tetraethyl orthosilicate (TEOS, 98%),* p*-toluenesulfonyl chloride (TsCl), ADA, doxorubicin (DOX, 99%), mPEG (Mw 5000), DTT, 1-ethyl-3-(3-dimethylaminopropyl) carbodiimide (EDC, 97%), N,N-dimethyl formamide (DMF), and ethylenediamine (EDA, 99%) were purchased from Sigma-Aldrich (St. Louis, MO, USA). Cetyltrimethylammonium bromide (CTAB, 99%) was purchased from Merck (USA). DTDP (99%), 3-aminopropythiethoxysilane (APS, 99%), and 4-nitrophenyl chloroformate (PNC, 97%) were purchased from Acros Organics (Geel, Belgium). *β*-CD was purchased from TCI Co. (Tokyo, Japan). All chemicals were used as received without further purification.

### 2.2. Preparation of PNS-SS-ADA

According to the literature with minor modification, the overall process of PNS-SS-ADA preparation can be described in four main steps [7]. (1) the synthesis of PNS was performed by sol-gel method. Briefly, deionized water (diH_2_O, 64 mM), ethanol (0.2 mol), CTAB (7.1 mmol), and 2.8% NH_3_ solution (0.9 mmol) were mixed at 60°C under stirring for 30 min. TEOS solution (35.8 mmol) was added dropwise into the surfactant solution under constant stirring, and the reaction was continued for 2 h and then filtered. The filtrate was dialyzed using a dialysis membrane (MWCO 6–8 kDa, Spectrum Laboratories, Inc., USA) against diH_2_O for 4 days at room temperature and later lyophilized to collect PNS. (2) The preparation of PNS-NH_2_ was carried out by mixing APS (5.7 mmol) and PNS (1 g) dissolved in methanol (4 mL); the reaction was kept at 30°C for 8 h before starting sonication for 30 min. Thus, the solution was dialyzed for 4 days against 2 M acetic acid, ethanol (1 v/v, 250 mL), and then immersed into diH_2_O for a day. The solution was freeze-dried to form PNS-NH_2_ as a white powder. (3) The prepared PNS-NH_2_ (1 g) and EDC (0.14 mL) were dissolved together into diH_2_O (20 mL) for 10 min. Thereafter, DTDP (0.77 mmol) dissolved in 20 mL of DMF was added to the mixture and the reaction was maintained for 24 h. The samples were purified by a dialysis membrane (MWCO 6–8 kDa) against diH_2_O at room temperature for 4 days, followed by lyophilizaytion to obtain PNS-SS-COOH. (4) The obtained PNS-SS-COOH (1 g) and ADA solution (0.77 mmol) were mixed together under stirring, followed by the addition of EDC (0.64 mmol). The reaction was stirred at room temperature for 2 h and filtered. The sample was dialyzed at room temperature and finally lyophilized to get PNS-SS-ADA.

### 2.3. Preparation of CD-mPEG Conjugate and DOX/PNS-SS-ADA@CD-mPEG

The synthesis of CD-mPEG was carried out in four main steps [7]: (1) mPEG-NH_2_ was fabricated under controlled temperature and vacuum conditions. Initially, mPEG (0.16 mmol) was melted down at 65°C under vacuum. Thus, PNC (0.19 mmol) was added to the above mPEG solution under constant stirring for 6 h, followed by the addition of 20 mL of THF solution. The obtained mPEG-PNC was gently dropped into EDA solution (0.23 mmol) and the mixture was stirred at room temperature for 24 h. The solution was dialyzed using dialysis membrane (MWCO 3.5 kDa) and then lyophilized to obtain mPEG-NH_2_. (2) *β*-CD (26.4 mmol) and 10 mL of NaOH solution (8.2 M) were both dissolved in diH_2_O (200 mL) under constant stirring for 10 min. TsCl (26.4 mmol) dissolved in 15 mL of acetonitrile was added to the mixture and the reaction was maintained at 25°C for 2 h. The pH value of the solution was adjusted to 8.0. The solid product was collected at 5–8°C for 24 h, achieved under vacuum at room temperature, and dried at 70°C for 2-3 days. (3) In order to synthesize CD-NH_2_, CD-OTs (1 mmol) was dispersed in 30 mL of DMF under stirring condition, followed by the addition of EDA (30 mmol). The reaction was carried out for 24 h at 75°C under conditions of stirring and nitrogen flushing. The mixture was evaporated and precipitated in acetone solution (500 mL) for several times and, in addition, the solid product was later dried under vacuum to obtain CD-NH_2_. (4) Both mPEG-NH_2_ and CD-NH_2_ were used with the support of EDC chemistry to obtain CD-mPEG. In detail, CD-NH_2_ (7.4 *μ*mol) and EDC (0.09 mmol) were dissolved in 10 mL of diH_2_O under constant stirring, followed by the addition of mPEG-NH_2_ (0.1 mmol) into the above mixture. After 24 h, the solution was filtered and dialyzed (MWCO 6–8 kDa) for 4 days and finally freeze-fried to collect CD-mPEG for further synthesis.

DOX was encapsulated into the PNS-SS-ADA nanoparticle by a sonication method. First, 5 mg of DOX and 20 mg of PNS-SS-ADA were stirred in diH_2_O and sonicated for 10 min. Next, CD-mPEG solution was added to the mixture, sonicated for 10 min, and stirred overnight. The sample was further purified by dialysis membrane (MWCO 3.5 kDa) against diH_2_O for 3 h and then lyophilized.

To prepare the empty PNS-SS-ADA@CD-mPEG nanoparticles, the same procedure was employed except the addition of DOX.

### 2.4. Characterization

Bruker Avance 500 (Bruker Co., USA) was used to record proton NMR spectroscopy of PNS-SS-ADA and CD-mPEG using D_2_O as a solvent. FTIR spectra of PNS, PNS-SS-ADA, and PNS-SS-ADA@CD-mPEG were recorded on a Bruker Equinox 55 FTIR (Bruker Co., USA) in order to investigate the presence of SS-ADA and CD-mPEG on the surface of PNS nanoparticles. TGA was carried out using TG Analyzer (Perkin Elmer Pryris 1, USA). N_2_ adsorption-desorption isotherms were measured using a NOVA 1000e system (Quantachrome Instruments, USA). The samples were outgassed for 3 h at 150°C before the measurements. Morphology and size of PNS and PNS-SS-ADA@CD-mPEG nanoparticles were imaged by TEM using JEM-1400 (JEOL, Tokyo, Japan) at an accelerating voltage of 300 kV. The samples for TEM observations were prepared by placing a drop of solution in diH_2_O (1 mg/mL) onto a carbon-copper grid (300 mesh, Ted Pella, Inc., USA) and air-dried for 10 min.

### 2.5. DOX Loading Contents and* In Vitro* DOX Release

The DOX-loaded PNS-SS-ADA@CD-mPEG (DOX/PNS-SS-ADA@CD-mPEG) nanocarriers were prepared using equilibrium dialysis. The DOX loading efficiency (DLE) and DOX loading capacity (DLC) were quantified indirectly from the amount of unloaded DOX (*W*_U-DOX_), which was evaluated using a UV-Vis spectrophotometer (NIR-V670, JASCO, Japan). Initially, fresh DOX was stirred in dH_2_O to obtain a series of different known concentrations (0–60 *μ*g/mL), which were used as standard samples. The absorbance of the standard and dialysis solutions was recorded at 495 nm using a V-750 UV/Vis spectrophotometer (Jasco Co., Tokyo, Japan). The following equations were used to calculate the DLE and DLC:

(1) DLE%=5−WU-DOX5∗100,DLC%=5−WU-DOXWPNS-SS-ADA@CD-mPEG+5−WU-DOX∗100,

where 5 mg is the initial amount of DOX for loading experiment; *W*_U-DOX_ is the total amount of unloaded DOX in the dialysis solution; *W*_PNS-SS-ADA@CD-mPEG_ is the dried weight of polymer in the nanocarrier.

DOX release kinetics from DOX/PNS-SS-ADA@CD-mPEG nanocarriers were conducted in PBS (10 mM, pH 7.4). Each sample (1 mL) was independently transferred into dialysis bags (MWCO 3.5 kDa). These sample bags were immersed in fresh media (14 mL, PBS, and pH 7.4) under constant stirring at 37°C. At specific time intervals, the release medium (14 mL) was collected and an equivalent amount of fresh medium was added. The amount of DOX released was determined using UV-Vis spectrophotometer as mentioned previously [20], especially, the release of DOX from DOX/PNS-SS-ADA@CD-mPEG under reducing condition (5 mM DTT).

### 2.6. MTT Viability Test

In order to evaluate the cytotoxicity of DOX/PNS-SS-ADA@CD-mPEG, the MTT assay was carried out. HeLa cells (ATCC, Manassas, VA, USA) were seeded in wells of 96-well plates (Sigma-Aldrich Co., St. Louis, MO, USA) with a density of 1 × 10^4^ cells/well in 130 *μ*L of DMEM (Dulbecco's Modified Eagle's medium, Acros Organics, Geel, Belgium) supplemented with 10% Fetal Bovine Serum (FBV) and 1% penicillin-streptomycin, and cultured at 37°C for a day. The media were removed, and thus the cells were incubated with media containing various sample concentrations for later 48 h, followed by removing media and washing twice with PBS.

MTT solution (25 *μ*L, 2 mg/mL) and culture medium (130 *μ*L) were added to each well. The cells were cultured for further 3 h, followed by adding DMSO (130 *μ*L) into each well to dissolve the precipitated purple formazan in 15 min. The samples were then transferred into new transparent 96-wells plates and the absorbance at 570 nm was recorded using a multiplate reader (SpectraMax M2E, Molecular Devices Co., USA).

### 2.7. Statistical Analysis

The data were expressed as mean ± standard deviation. The statistical data evaluation was performed using Microsoft® Excel.

## 3. Results and Discussion

### 3.1. Characterization of PNS-SS-ADA@CD-mPEG

**Figure 2:**
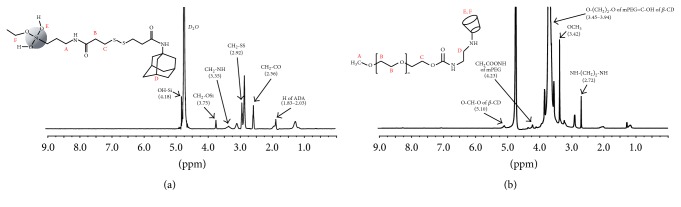
^1^H NMR spectra: PNS-SS-ADA (a); CD-mPEG (b).

PNS nanoparticles were synthesized using the sol-gel technique reported in previous literature [21].  Figure 2 shows the proton NMR with assigned protons of PNS-SS-ADA and CD-mPEG. The peak at 4.67 ppm corresponded to D_2_O as a solvent. As shown in Figure 2(a), protons at 3.35 ppm (peak A), 2.56 ppm (peak B), 2.92 ppm (peak C), 1.83–2.03 ppm (peak D), 4.18 ppm (peak E), and 3.75 ppm (peak F) were assigned to CH_2_-NH, CH_2_-CO, CH_2_-SS, H of ADA, OH-Si, and CH_2_-OSi groups, respectively. The presence of all these signals indicated the successful preparation of PNS-SS-ADA.

The ^1^H NMR spectrum of CD-mPEG conjugate presents peaks at 3.42 ppm (peak A), 3.45–3.94 ppm (peak B and E), 4.23 ppm (peak C), 5.10 ppm (peak F), and 2.72 ppm (peak D), which corresponded to OCH_3_, O-(CH_2_)_2_-O of mPEG skeleton and =C-OH of *β*-CD, CH_2_COONH of mPEG, O-CH-O of *β*-CD, and NH-(CH_2_)_2_-NH, respectively. These results indicated the success of the synthesis of CD-mPEG.

**Figure 3:**
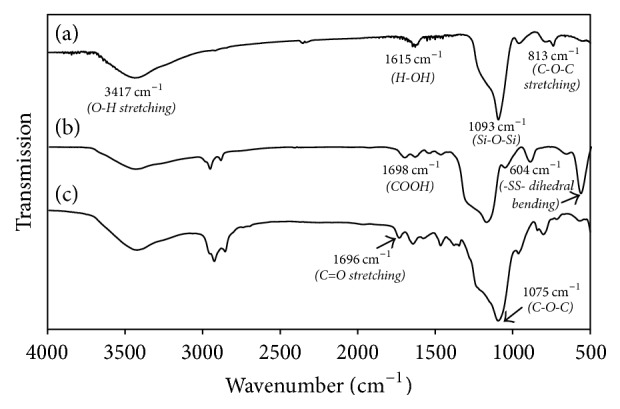
FTIR spectra: PNS (a) [7]; PNS-SS-ADA (b) [7]; PNS-SS-ADA@CD-mPEG (c).

In addition, the successful conjugation of CD with mPEG was evaluated by FTIR analysis (Figure 3). As shown in the FTIR spectrum of PNS (Figure 3(a)), a band at 1615 cm^−1^ was attributed to the OH bending vibration of water molecules on the surface of PNS. The large sharp peak at 1093 cm^−1^ and peak at 813 cm^−1^ were both assigned to the stretching modes of the siloxane framework, which is the asymmetric stretching frequency of Si-O-Si. Additionally, a broad band observed at 3417 cm^−1^ was associated with OH stretching frequency for the silanol group. These adsorption bands still exist in the FTIR spectra of PNS-SS-ADA (Figure 3(b)) and PNS-SS-ADA@CD-mPEG (Figure 3(c)). When PNS was immobilized with ADA, the characteristic absorption band at 1698 cm^−1^ was attributed to the adsorption of carboxyl group and two new bands of amantadine, indicating the successful conjugation between ADA and PNS [7]. Figure 3(c) shows the intensity of IR peak appeared at 604 cm^−1^, which was attributed to –SS- dihedral bending of DTDP. The visible absorption peaks at 1696 cm^−1^ and 1608 cm^−1^ were ascribed to the C=O stretching frequency (amide I) and N-H bending vibration in alkyl chains of mPEG. These results suggested that PNS-SS-ADA@CD-mPEG was prepared [7].

### 3.2. Particle Properties of PNS-SS-ADA@CD-mPEG

**Figure 4:**
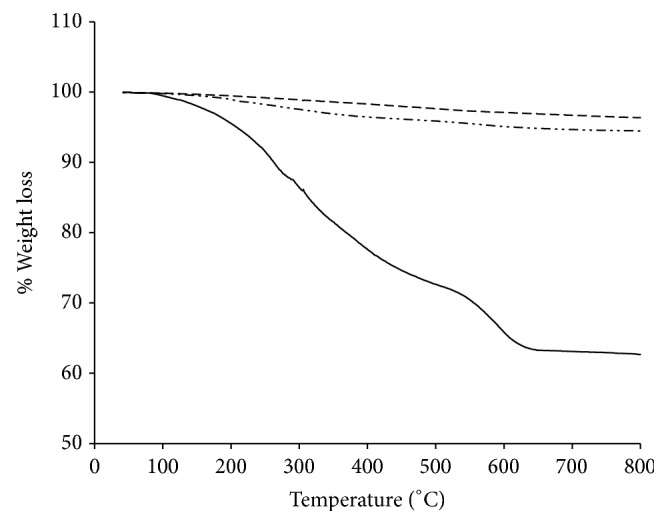
TGA curves: PNS* (dashed curve)*; PNS-SS-ADA* (double dashed dotted curve)*; PNS-SS-ADA@CD-mPEG* (solid curve)*.

The typical TGA curves of PNS (dotted curve), PNS-SS-ADA (dash-dotted curve), and PNS-SS-ADA@CD-mPEG (solid curve) are shown in Figure 4. The temperature was ramped up 800°C with a heating rate at 10°C/min. As shown in the TGA curve of PNS, only 3.33% weight loss was observed caused by the loss of physisorbed water. There is no statistically significant difference between the weight loss of PNS and PNS-SS-ADA, approximately 2.27%, which may be related to the thermal decomposition of outer layer SS-ADA. By contrast, the total weight loss of PNS-SS-ADA@CD-mPEG was 57.33%, which was much higher than that of PNS (3.33%) and PNS-SS-ADA (5.60%). This increased portion could be attributed to the CD-mPEG conjugate. Furthermore, there remained 42.67% of weight that may be due to the PNS residue, suggesting that the surface of PNS-SS-ADA was immobilized with CD-mPEG.

**Figure 5:**
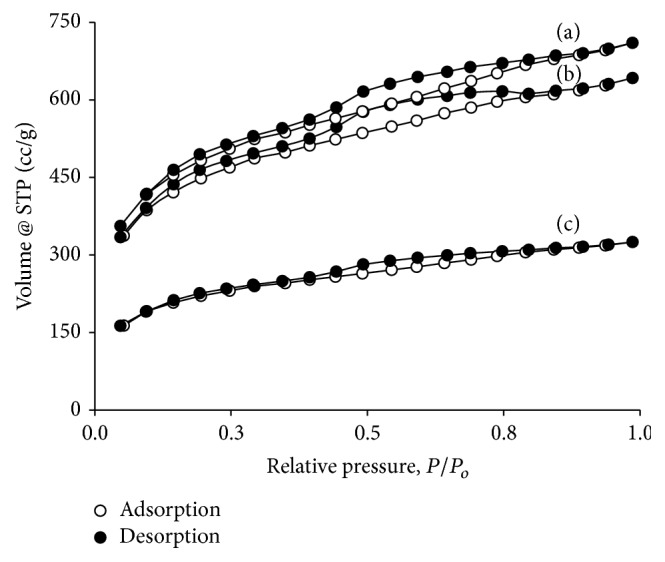
N_2_ adsorption-desorption isotherms: PNS (a); PNS-SS-ADA (b); PNS-SS-ADA@CD-mPEG (c).

The N_2_ adosrption-desorption isotherms of PNS, PNS-SS-ADA, and PNS-SS-ADA@CD-mPEG are shown in Figure 5. These isotherms exhibit a type IV hysteresis loop with type H_2_ hysteresis loops at a relative pressure *P*/*P*_*o*_ = 0.4–0.8, which is caused by capillary condensation of mesoporous adsorption [22]. There were significant differences in pore volume (*V*_*p*_, cm^3^/g) among PNS, PNS-SS-ADA, and PNS-SS-ADA@CD-mPEG, which are 710.40 cm^3^/g, 642.20 cm^3^/g, and 324.87 cm^3^/g, respectively. The reduced *V*_*p*_ of the modified PNS indicated that the mesoporous structure of PNS was affected by surface modification. These results could be explained by the presence of the outer layer on the surface of PNS nanoparticles.

**Figure 6:**
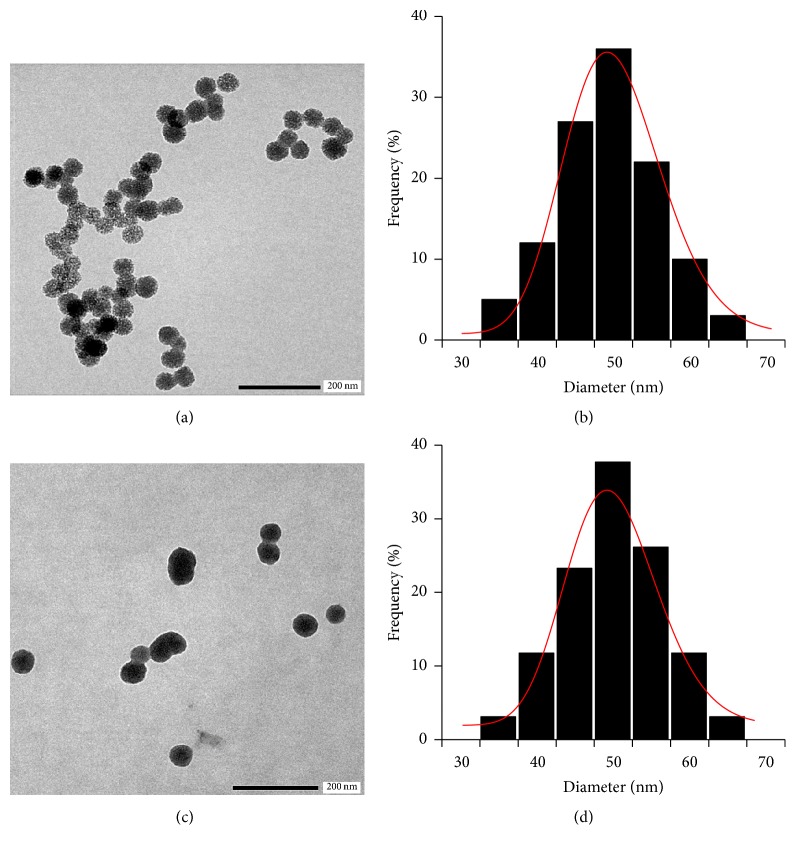
TEM images and particle size histograms: PNS (a, b); PNS-SS-ADA@CD-mPEG (c, d) fitted by log-normal distribution function, respectively.

The TEM image and typical particle size histogram fitted by long normal distribution function of PNS and PNS-SS-ADA@CD-mPEG are shown in Figure 6. The morphology of both nanoparticles was spherical in shape, and their particle sizes were also different, 49.6 ± 2.56 nm for PNS and 55.5 ± 3.05 nm for PNS-SS-ADA@CD-mPEG. This difference was attributed to the presence of outer layer SS-ADA@CD-mPEG on the surface of PNS nanoparticles. Previous studies have indicated that nanoparticles less than 20–30 nm are rapidly eliminated by renal excretion, whereas nanoparticles in the range of 30–150 nm can stay in the circulatory system for a longer period of time [23]. One of the most critical insights of PNS is that the bioactive molecules can be significantly loaded into the porous pores of silica nanoparticles. More importantly, the release of DOX from DOX/PNS-SS-ADA@CD-mPEG can be controlled and prolonged due to the presence of SS-ADA@CD-mPEG. These results suggest that the modified PNS-SS-ADA@CD-mPEG nanoparticles have great potential as an efficient and promising candidate for controlling drug release.

### 3.3. Loading and* In Vitro* DOX Release

The DOX-loaded formulations were prepared using equilibrium dialysis technique. The DLE and DLC of PNS-SS-ADA@CD-mPEG were found to be 79.2 ± 3.2% and 13.8 ± 2.7%, respectively. These results might be due to the loading of DOX molecules into the PNS-SS-ADA nanoparticles before CD-mPEG conjugation, which prevents the diffusion of DOX to the solution. These findings also demonstrated that PNS-SS-ADA@CD-mPEG nanoparticles have the potential to increase drug encapsulation.

**Figure 7:**
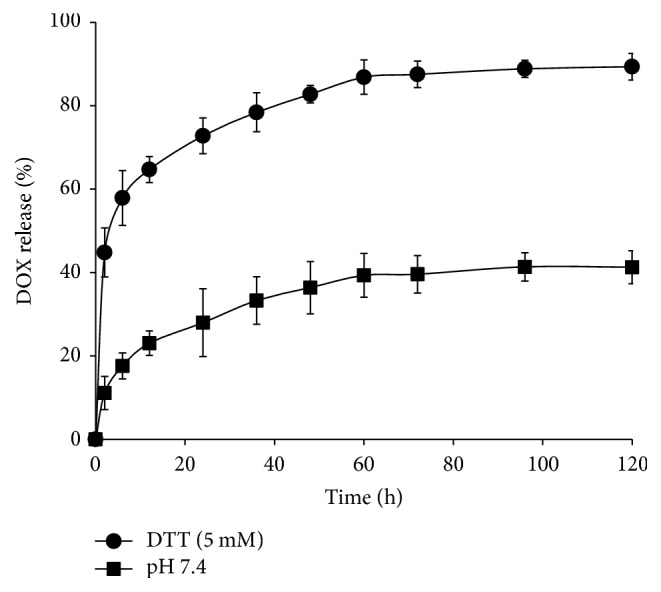
*In vitro* release profiles of DOX from PNS-SS-ADA@CD-mPEG in PBS (pH 7.4) with and without DTT (5 mM).

To further evaluate the redox responsiveness of the modified PNS-SS-ADA@CD-mPEG nanoparticles,* in vitro* release profile of DOX from DOX/PNS-SS-ADA@CD-mPEG nanocarriers was performed under reducing condition of 5 mM DTT. The release rate of the nanocarriers is expected to increase by the addition of DTT-reducing agent used to break down disulfide bonds of DOX/PNS-SS-ADA@CD-mPEG nanoparticles [7, 24, 25].  Figure 7 shows the DOX release behavior of DOX/PNS-SS-ADA@CD-mPEG nanoparticles with and without DTT over a period of 120 h. It can clearly be seen that, in the presence of DTT, the release of DOX from DOX/PNS-SS-ADA@CD-mPEG was significantly faster than that under the physiological condition (pH 7.4). During the first 2 h, the cumulative release amount of DOX was approximately 39.6% as compared with 10.7% in the pH 7.4 PBS buffer. Furthermore, around 89.3% of the total drug amount was released after 120 h for DTT condition; by contrast, 44.3% of DOX was observed within the same time frame in the absence of DTT. The DOX-loaded formulation showed a long-term stable drug release profile up to 120 h in PBS condition (pH 7.4) and a redox-responsive drug release behavior in the presence of DTT. These results indicated the functionalization of disulfide bonds on the surface of PNS, which might undergo swift oxidation and accelerate the DOX release rate in the intracellular region.

### 3.4. *In Vitro* Cytotoxicity

**Figure 8:**
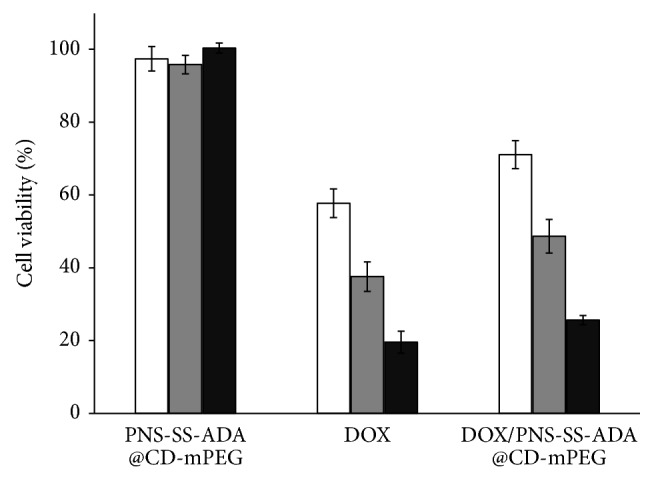
Viability of HeLa cells incubated with PNS-SS-ADA@CD-mPEG (100 *μ*g/mL), free DOX (5 *μ*g/mL), and DOX/PNS-SS-ADA@CD-mPEG (eq. DOX conc.) for 24, 48, and 96 h. The cells were exposed to the samples for the indicated times. The data represent the mean values ± the standard deviation (SD) (*n* = 4).

The biocompatibility of nanomaterials plays a crucial role in their potential biomedical applications [26, 27]. In this study, the* in vitro* cytotoxicity effect of the prepared PNS-SS-ADA@CD-mPEG, free DOX,and DOX/PNS-SS-ADA@CD-mPEG against HeLa cells was determined. As shown in Figure 8, PNS-SS-ADA@CD-mPEG exhibited cytocompatibility towards the cells with approximately 98% cell viability at 100 *μ*g/mL for 96 h. In contrast, free DOX showed acute toxicity to the cell line; the cell viability dropped to less than 20% after 96 h. The DOX/PNS-SS-ADA@CD-mPEG showed the improvement in the cytocompatibility compared with DOX alone, at 70% cell viability for 24 h. The lower cytotoxicity of DOX/PNS-SS-ADA@CD-mPEG nanoparticles can be attributed to their extended drug release characteristic. These results confirmed the promising application of PNS-SS-ADA@CD-mPEG nanocarriers with low systemic toxicity for controlled drug delivery.

## 4. Conclusion

In this report, PNS-SS-ADA@CD-mPEG nanoparticles have been successfully prepared via the conjugation between PNS-SS-ADA and CD-mPEG. The formed PNS-SS-ADA@CD-mPEG existed in a spherical shape with an average diameter of 55.5 ± 3.05 nm and acted as potential nanocarriers for effectively loading and controlled release of DOX. The prepared PNS-SS-ADA@CD-mPEG nanocarriers showed a high cell viability against HeLa cancer cells. DOX was significantly encapsulated into the formed PNS-SS-ADA@CD-mPEG nanoparticles, at 79.2 ± 3.2%. Moreover, the release of DOX from DOX/PNS-SS-ADA@CD-mPEG nanocarriers was controlled and delayed up to 120 h in PBS buffer (pH 7.4), compared with less than 40 h in buffer with 5 mM DTT. The developed PNS-SS-ADA@CD-mPEG nanoparticle could be a potential candidate for controlling the release of anticancer drugs with high drug loading efficiency and less systemic cytotoxicity.
